# Physiologically Based Pharmacokinetic (PBPK) Model of Gold Nanoparticle-Based Drug Delivery System for Stavudine Biodistribution

**DOI:** 10.3390/pharmaceutics14020406

**Published:** 2022-02-13

**Authors:** Hinojal Zazo, Clara I. Colino, Carmen Gutiérrez-Millán, Andres A. Cordero, Matthias Bartneck, José M. Lanao

**Affiliations:** 1 Area of Pharmacy and Pharmaceutical Technology, Faculty of Pharmacy, Avda Lcdo Méndez Nieto, 37007 Salamanca, Spain; hinojal@usal.es (H.Z.); carmengutierrez@usal.es (C.G.-M.); andresac@usal.es (A.A.C.); 2 Institute of Biomedical Research of Salamanca (IBSAL), 37007 Salamanca, Spain; 3 Department of Medicine III, Medical Faculty, RWTH Aachen, Pauwelsstr. 30, 52074 Aachen, Germany; mbartneck@ukaachen.de

**Keywords:** gold nanoparticles, stavudine, pharmacokinetics, biodistribution, PBPK model

## Abstract

Computational modelling has gained attention for evaluating nanoparticle-based drug delivery systems. Physiologically based pharmacokinetic (PBPK) modelling provides a mechanistic approach for evaluating drug biodistribution. The aim of this work is to develop a specific PBPK model to simulate stavudine biodistribution after the administration of a 40 nm gold nanoparticle-based drug delivery system in rats. The model parameters used have been obtained from literature, in vitro and in vivo studies, and computer optimization. Based on these, the PBPK model was built, and the compartments included were considered as permeability rate-limited tissues. In comparison with stavudine solution, a higher biodistribution of stavudine into HIV reservoirs and the modification of pharmacokinetic parameters such as the mean residence time (MRT) have been observed. These changes are particularly noteworthy in the liver, which presents a higher partition coefficient (from 0.27 to 0.55) and higher MRT (from 1.28 to 5.67 h). Simulated stavudine concentrations successfully describe these changes in the in vivo study results. The average fold error of predicted concentrations after the administration of stavudine-gold nanoparticles was within the 0.5–2-fold error in all of the tissues. Thus, this PBPK model approach may help with the pre-clinical extrapolation to other administration routes or the species of stavudine gold nanoparticles.

## 1. Introduction

Nowadays, nanoparticle-based drug delivery systems are one of the most important research issues for the treatment of a wide range of pathologies, mainly due to their multifunctionality [1]. However, nanoparticle properties, including size, charge, and surface functionalization, affect tissue reservoir targeting and translate into different pharmacokinetic behaviors. These differences in their characteristics limit pre-clinical assessments compared with conventional formulations [2]. This problem can be solved by the use of mathematical and computational modelling, encompassing discrete models based on quantum mechanics, molecular dynamics, etc., or continuous ones, such as pharmacoki-netic/pharmacodynamic (PK/PD) modelling, physiologically based pharmacokinetic (PBPK) modelling, and so on [3].

Among all of these, PBPK modelling and simulation have been selected for the current research, because it provides a more mechanistic approach for studying drug disposition in individual organs and tissues, and even allows the prediction of human pharmacokinetics from preclinical research. In fact, this tool has gained attention in drug research and development, for both conventional [4,5] and nanoparticle formulations [6,7]. 

Human immunodeficiency virus (HIV) accumulates in macrophages, the spleen, and liver, where the viable virus replicates survive either as virions or as latent, infected cells, and from where they spread throughout the body. So, therapeutic intracellular levels into these tissue reservoirs are necessary to eradicate the virus [8]. The use of drug delivery systems to treat this infection allows antiretroviral drugs to be specifically targeted to reservoirs. This may be achieved by using nanoparticles that are quickly recognized by mononuclear phagocyte cells, such as macrophages, and accumulate in the spleen and liver [3,9].

Stavudine is an antiretroviral drug that presents dose-dependent side effects, very short half-life, and low manufacturing costs [10]. Hence, different kinds of nanosystems have been used as carriers for stavudine [11,12,13,14]. 

Metallic nanosystems such as gold nanoparticles (AuNP) are promising because they can be synthesized easily and exhibit a unique combination of physical, chemical, optical, and electronic properties, in comparison with other biomedical nanotechnologies. Moreover, conjugation with multiple and different molecules is possible due to their small size, so they can be used as drug delivery systems transporting a huge number of molecules [2,15]. Because of this, gold stavudine nanocarriers have previously been proposed by our group for the macrophage-targeted therapy of HIV [16].

In recent years, different PBPK models of nanoparticles, alone or as drug carriers, have been published, addressing different aspects of the pharmacokinetic properties of nanoparticles [6,7]. However, very few of them are related to anti-HIV drugs or the gold nanoparticle drug delivery systems of anti-HIV drugs. In fact, as far as we know, no one is focused on gold nanoparticles as a drug delivery system of anti-HIV drugs.

The aim of this work was to develop a PBPK model that simulates the biodistribution of stavudine after its administration in a gold nanoparticle-based drug delivery system, integrating in vitro and in vivo study results. 

## 2. Materials and Methods 

### 2.1. Materials

Stavudine was obtained from ArQuifar (Barcelona, Spain), and 40 nm AuNPs were stabilised with citrate from NanoComposix (San Diego, CA, USA). The human blood for macrophage isolation was obtained from Transfusion Medicine of RWTH Aachen University Hospital (Aachen, Germany). Male Wistar rats were supplied by the NUCLEUS Service of the University of Salamanca (Experimental Animal Service). The housing and experimental treatment of animals was in accordance with current Spanish (RD 1386/2018, BOE 20/11/2018) and European Union (2010/63/UE) legislation, and complied with “Principles of Laboratory Animal Care”. Protocols used in this study have been approved by the Committee of Bioethics of the University of Salamanca. 

### 2.2. Development of Stavudine Gold Nanoparticles 

Nanocarriers were developed following the same methods as in our previous work [16]. Moreover, 1 mL of AuNP (0.05 mg Au/mL) sizing 40 nm (Z potential −51.63 ± 0.50) was incubated with 0.5 mL of stavudine solution of 3 mg/mL in a shaking bath at room temperature (20 °C) for 24 h. Then, the suspension was centrifuged at 18,000× *g* for 15 min to separate stavudine-AuNP from the watery solution. Stavudine loading efficacy was calculated by an indirect method from stavudine-free concentrations in the supernatant quantified by a UHPLC-UV method.

- Drug Release Kinetics

The drug release study was done in PBS under two different pH conditions (7.4 and 5.5). First, 100 µL of stavudine-AuNP solution was suspended in 1 mL of PBS. This solution was kept at 37 °C with vigorous shaking until 72 h. At different times (1, 24, 48, and 72 h), samples were taken and centrifuged at 18,000× *g* for 15 min, and finally, the supernatant drug concentration was analysed by UHPLC-UV. The percentage of drug released was calculated from the UHPLC results, taking into account the amount of loaded drug. These results were fitted by non-linear regression to different release models. The final model was selected based on goodness-of-fit criteria. This kind of information was used in the building of the PBPK model.

### 2.3. Uptake Kinetics in Cells 

The in vitro uptake kinetic study of stavudine solution and stavudine AuNPs by cells was studied in order to be used subsequently in the PBPK model. The cells used were differentiated macrophages from human peripheral blood mononuclear cells (PBMC). They were isolated from human blood, using Ficoll-based density gradient centrifugation, as described earlier [16]. Briefly, PBMC were incubated in RPMI 1640, with 5% human autologous serum on uncoated Petri dishes (2 million PBMS/mL), for 35 min, at 37 °C and 5% CO_2_. During this period, monocytes adhered to the dish, and subsequently, lymphocytes were removed with the supernatant. To obtain human primary macrophages, monocytes were cultured for seven days in RPMI1640 medium supplemented with 5% autologous human serum. Once isolated, macrophages were incubated with 10 µL of 10 µg/mL of stavudine, from solution or stavudine-AuNP. After 1, 6, 24 and 48 h of incubation, samples were harvested by brushing the bottom of the plate after being refrigerated for 10 or 15 min [16]. Drug content was measured by UHPLC analysis, and the percentage uptake calculations were done considering the 10 µg/mL of stavudine added as 100%.

### 2.4. In Vivo Pharmacokinetic Study 

For in vivo assays, 38 male Wistar rats, with a mean weight of 252 ± 12 g, were used. Animals were treated intraperitoneally with 1 mL of the drug formulation. In the control group, the stavudine solution mean dose was 11.9 ± 0.5 mg/kg. In the AuNP group, the stavudine mean dose was 15.6 ± 0.6 mg/kg loaded in gold nanoparticles, with a mean dose of 0.98 ± 0.05 mg Au/kg. Animals were sacrificed at different times (1, 3, 6, 12, and 24 h) after drug administration. Samples of plasma, liver, spleen, thymus, and brain were obtained.

### 2.5. Quantification of Stavudine by UHPLC Analysis 

The quantification of stavudine in the nanoparticle supernatant, cells, plasma and tissues was done with a previously described method adapted to ultra-high pressure liquid chromatography (UHPLC) [17]. The chromatographic separation was performed in a Kinetex^®^ C_18_ column (50 mm × 2.10 mm, particle size 1.7 μm, Phenomenex^®^) at 45 °C. The mobile phase was water: acetonitrile (94:6 *v/v*) at a flow rate of 0.50 mL/min. A Shimadzu UHPLC (Tokyo, Japan) with a PDA SPD-M20A detector (Shimadzu, Tokyo, Japan) and a 265 nm wavelength was used for detection. 

To quantify intracellular stavudine concentration, cell culture samples were treated with 20% trichloroacetic acid (TCA) in a ratio 2:1 (*v/v*) sample:TCA. Samples were centrifuged at 18,000× *g* for 15 min and supernatants were analysed.

Tissue samples were homogenized in 0.5 mL/g of 6.7 × 10^−2^ M phosphate buffer, pH 7.4, plus 0.5 mL/g of 0.25 mM Triton X-100, using a Pro 250 homogenizer. Homogenates and plasma samples were mixed with 30% TCA (10:1 *v/v*), to precipitate the proteins, and centrifuged at 14,000× *g* for 5 min. 

All samples were filtered by a 0.22 µm pore diameter nylon membrane before being analysed.

The stavudine concentrations were determined by an external standard method. The inter-day and intra-day variabilities of the instrument for aqueous samples were below 5%. For biological samples, the method’s inter-day and intra-day variabilities were below 15%, and the quantification limit was 1 ng/mL.

### 2.6. Kinetic Analysis

#### 2.6.1. Model-Independent Analysis of In Vivo Study Results

In vivo data were characterised using model-independent pharmacokinetic analysis [18]. The calculated pharmacokinetic parameters were: total area under the curve (${\mathrm{AUC}}_{\infty }^{0}$), plasma clearance (*Cl*), apparent distribution volume (*Vd*), mean residence time (MRT), and plasma or tissue half-life (t_1/2_).

#### 2.6.2. PBPK Model

- Model building

**Figure 1:**
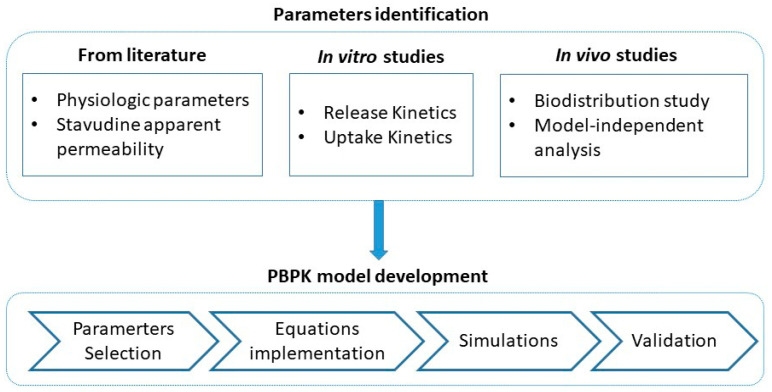
Workflow of the model building procedure.

The development of the PBPK model starts by identifying parameters related to stavudine and its formulation to be used in the model. Due to the fact that some parameters were not available in the literature, the next step was carrying out in vitro and in vivo assays in order to have specific values for this formulation. Once all parameters were available, the PBPK model was built. Figure 1 shows the flowchart with the different stages used in building the PBPK model.

**Figure 2:**
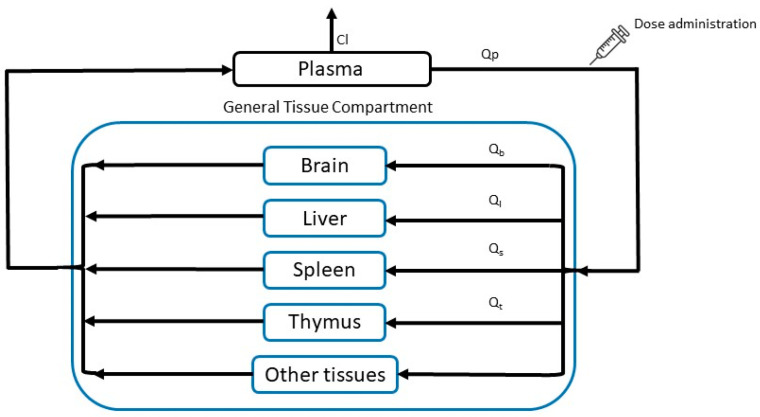
PBPK model structure after instantaneous administration. Cl, clearance; Qi, blood flow of each tissue.

The whole-body PBPK biodistribution model for stavudine solution and stavudine-AuNP consisted of plasma, a general tissue compartment, and relevant specific organs, such as the liver, spleen, thymus, and brain. All tissues that were not sampled were included in the “other tissues” compartment (Figure 2). Elimination was represented by free drug clearance. Drug concentrations in each tissue were determined by mass balance equations. All tissues included in the model have been considered permeability-limited, which means that each tissue compartment was divided into two sub-compartments, vascular and extravascular, separated by a cell membrane barrier. The cell membranes were considered as diffusional barriers to the studied molecule, with a bidirectional perfusion for the free drug and unidirectional uptake process for nanoparticles [6]. 

**Figure 3:**
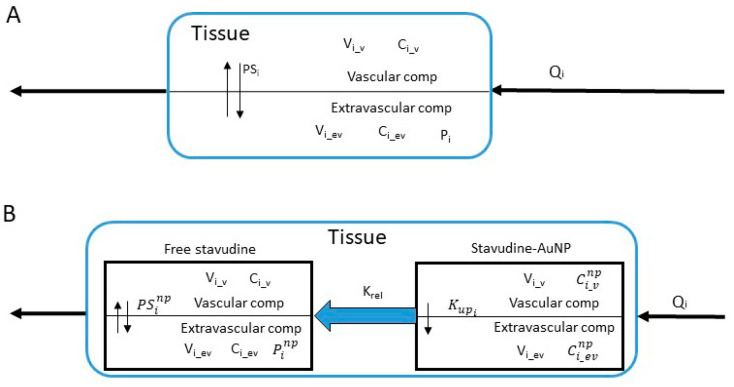
Tissue structure for stavudine solution (**A**) and stavudine-AuNP (**B**). *C_i_v_* and *C_i_ev_*, stavudine vascular and extravascular concentration of each tissue; $C_{i\_v}^{np}$ and $C_{i\_ev}^{np}$ stavudine-AuNP vascular and extravascular concentration of each tissue; $K_{up_{i}}$, AuNP uptake rate constant estimated of each tissue; *P_i_* and $P_{i}^{np}$, partition coefficient of each tissue with and without nanoparticles; *PS_i_* and $PS_{i}^{np}$ permeability-surface area coefficient of each tissue with and without nanoparticles; *Q_i_*, blood flow of of each tissue; *V_i_v_* and *V_i_ev_*, vascular and extravascular volume of each tissue.

Two PBPK models were developed, one for stavudine solution and another for stavudine-AuNP administration. Considering the fast absorption of drugs after intraperitoneal administration, and to simplify the PBPK model, the administration route used for the in vivo study was described as an instantaneous administration in the model [19]. Both models had the same structure (Figure 2), though in the stavudine-AuNP PBPK model, each tissue included another compartment, from which the free drug was released (Figure 3).

- Model Equations

PBPK model equations have been adapted from Nestorov I. [20] and Espié P. et al. [21]. In the following, equations for the simulation of stavudine plasma and tissue levels after administration as stavudine solution are shown:

- Plasma
  (1) $$Vd\times \frac{dC_{p}}{dt}=Q_{p}\times \left(\frac{C_{gt}}{P_{gt}}-C_{p}\right)-Cl\times C_{p}$$
- General Tissue
  (2) $$Vd\times \frac{dC_{gt}}{dt}=Q_{p}\times \left(C_{p}-\frac{C_{gt}}{P_{gt}}\right)$$
- Brain
  ○ Vascular compartment
    (3) $$V_{b\_v}\times \frac{dC_{b\_v}}{dt}=Q_{b}\times \left(C_{p}-C_{b\_v}\right)+PS_{b}\times \left(\frac{C_{b\_ev}}{P_{b}}-C_{b\_v}\right)$$
  ○ Extravascular compartment
    (4) $$V_{b\_ev}\times \frac{dC_{b\_ev}}{dt}=PS_{b}\times \left(C_{b\_v}-\frac{C_{b\_ev}}{P_{b}}\right)$$
- Liver
  ○ Vascular compartment
    (5) $$V_{l\_v}\times \frac{dC_{l\_v}}{dt}=Q_{l}\times \left(C_{p}-C_{l\_v}\right)+PS_{l}\times \left(\frac{C_{l\_ev}}{P_{l}}-C_{l\_v}\right)$$
  ○ Extravascular compartment
    (6) $$V_{l\_ev}\times \frac{dC_{l\_ev}}{dt}=PS_{l}\times \left(C_{l\_v}-\frac{C_{l\_ev}}{P_{l}}\right)$$
- Spleen
  ○ Vascular compartment
    (7) $$V_{s\_v}\times \frac{dC_{s\_v}}{dt}=Q_{s}\times \left(C_{p}-C_{s\_v}\right)+PS_{s}\times \left(\frac{C_{s\_ev}}{P_{s}}-C_{s\_v}\right)$$
  ○ Extravascular compartment
    (8) $$V_{s\_ev}\times \frac{dC_{s\_ev}}{dt}=PS_{s}\times \left(C_{s\_v}-\frac{C_{s\_ev}}{P_{s}}\right)$$
- Thymus
  ○ Vascular compartment
    (9) $$V_{t\_v}\times \frac{dC_{t\_v}}{dt}=Q_{t}\times \left(C_{p}-C_{t\_v}\right)+PS_{t}\times \left(\frac{C_{t\_ev}}{P_{t}}-C_{t\_v}\right)$$
  ○ Extravascular compartment
    (10) $$V_{t\_ev}\times \frac{dC_{t\_ev}}{dt}=PS_{t}\times \left(C_{t\_v}-\frac{C_{t\_ev}}{P_{t}}\right)$$
    where subindices and abbreviations mean: *_b_*, brain; *_gt_*, general tissue; *_l_*, liver; *_p_*, plasma; *_s_*, spleen; *_t_*, thymus; *C_i_v_* and *C_i_ev_*, stavudine vascular and extravascular concentration; *Cl*, clearance; *P_i_*, partition coefficient of each tissue when drug is administered as solution; *PS_i_* permeability-surface area coefficient; *Q_i_*, blood flow of each tissue; *V_i_*, compartment volume of each tissue; *Vd*, distribution volume.

In the following, equations for the simulation of stavudine plasma and tissue levels after administration of stavudine-AuNP are shown:

- Plasma
  ➢ Stavudine-AuNP
    (11) $$Vd\times \frac{dC_{p}^{np}}{dt}=Q_{p}\times \left(C_{gt}^{np}-C_{p}^{np}\right)-\left(K_{rel}\times C_{p}^{np}\times Vd\right)-\left(K_{up_{p}}\times C_{p}^{np}\right)$$
  ➢ Free stavudine
    (12) $$Vd\times \frac{dC_{p}}{dt}=Q_{p}\times \left(\frac{C_{gt}}{P_{gt}}-C_{p}\right)+\left(K_{rel}\times C_{p}^{np}\times Vd\right)-Cl\times C_{p}$$
- General Tissue
  ➢ Stavudine-AuNP
    (13) $$Vd\times \frac{dC_{gt}^{np}}{dt}=Q_{p}\times \left(C_{p}^{np}-C_{gt}^{np}\right)-\left(K_{rel}\times C_{gt}^{np}\times Vd\right)$$
  ➢ Free stavudine
    (14) $$Vd\times \frac{dC_{gt}}{dt}=Q_{p}\times \left(C_{p}-\frac{C_{gt}}{P_{gt}}\right)+\left(K_{rel}\times C_{gt}^{np}\times Vd\right)$$
- Brain
  ➢ Stavudine-AuNP
    ○ Vascular compartment
      (15) $$V_{b\_v}\times \frac{dC_{b\_v}^{np}}{dt}=Q_{b}\times \left(C_{p}^{np}-C_{b\_v}^{np}\right)-(K_{up_{b}}\times C_{b\_v}^{np})-\left(K_{rel}\times C_{b\_v}^{np}\times V_{b\_v}\right)$$
    ○ Extravascular compartment
      (16) $$V_{b\_ev}\times \frac{dC_{b\_ev}^{np}}{dt}=(K_{up_{b}}\times C_{b\_ev}^{np})-\left(K_{rel}\times C_{b\_ev}^{np}\times V_{b\_ev}\right)$$
  ➢ Free stavudine
    ○ Vascular compartment
      (17) $$V_{b\_v}\times \frac{dC_{b\_v}}{dt}=Q_{b}\times \left(C_{p}-C_{b\_v}\right)+PS_{b}^{np}\times \left(\frac{C_{b\_ev}}{P_{b}^{np}}-C_{b\_v}\right)+\left(K_{rel}\times C_{b\_v}^{np}\times V_{b\_v}\right)$$
    ○ Extravascular compartment
      (18) $$V_{b\_ev}\times \frac{dC_{b\_ev}}{dt}=PS_{b}^{np}\times \left(C_{b\_v}-\frac{C_{b\_ev}}{P_{b}^{np}}\right)+\left(K_{rel}\times C_{b\_ev}^{np}\times V_{b\_ev}\right)$$
- Liver
  ➢ Stavudine-AuNP
    ○ Vascular compartment
      (19) $$V_{l\_v}\times \frac{dC_{l\_v}^{np}}{dt}=Q_{l}\times \left(C_{p}^{np}-C_{l\_v}^{np}\right)-(K_{up_{l}}\times C_{l\_v}^{np})-\left(K_{rel}\times C_{l\_v}^{np}\times V_{l\_v}\right)$$
    ○ Extravascular compartment
      (20) $$V_{l\_ev}\times \frac{dC_{l\_ev}^{np}}{dt}=(K_{up_{l}}\times C_{l\_ev}^{np})-\left(K_{rel}\times C_{l\_ev}^{np}\times V_{l\_ev}\right)$$
  ➢ Free stavudine
    ○ Vascular compartment
      (21) $$V_{l\_v}\times \frac{dC_{l\_v}}{dt}=Q_{l}\times \left(C_{p}-C_{l\_v}\right)+PS_{l}^{np}\times \left(\frac{C_{l\_ev}}{P_{l}^{np}}-C_{l\_v}\right)+\left(K_{rel}\times C_{l\_v}^{np}\times V_{l\_v}\right)$$
    ○ Extravascular compartment
      (22) $$V_{l\_ev}\times \frac{dC_{l\_ev}}{dt}=PS_{l}^{np}\times \left(C_{l\_v}-\frac{C_{l\_ev}}{P_{l}^{np}}\right)+\left(K_{rel}\times C_{l\_ev}^{np}\times V_{l\_ev}\right)$$
- Spleen
  ➢ Stavudine AuNP
    ○ Vascular compartment
      (23) $$V_{s\_v}\times \frac{dC_{s\_v}^{np}}{dt}=Q_{s}\times \left(C_{p}^{np}-C_{s\_v}^{np}\right)-(K_{up_{s}}\times C_{s\_v}^{np})-\left(K_{rel}\times C_{s\_v}^{np}\times V_{s\_v}\right)$$
    ○ Extravascular compartment
      (24) $$V_{s\_ev}\times \frac{dC_{s\_ev}^{np}}{dt}=(K_{up_{s}}\times C_{s\_ev}^{np})-\left(K_{rel}\times C_{s\_ev}^{np}\times V_{s\_ev}\right)$$
  ➢ Free stavudine
    ○ Vascular compartment
      (25) $$V_{s\_v}\times \frac{dC_{s\_v}}{dt}=Q_{s}\times \left(C_{p}-C_{s\_v}\right)+PS_{s}^{np}\times \left(\frac{C_{s\_ev}}{P_{s}^{np}}-C_{s\_v}\right)+\left(K_{rel}\times C_{s\_v}^{np}\times V_{s\_v}\right)$$
    ○ Extravascular compartment
      (26) $$V_{s\_ev}\times \frac{dC_{s\_ev}}{dt}=PS_{s}^{np}\times \left(C_{s\_v}-\frac{C_{s\_ev}}{P_{s}^{np}}\right)+\left(K_{rel}\times C_{s\_ev}^{np}\times V_{s\_ev}\right)$$
- Thymus
  ➢ Stavudine-AuNP
    ○ Vascular compartment
      (27) $$V_{t\_v}\times \frac{dC_{t\_v}^{np}}{dt}=Q_{t}\times \left(C_{p}^{np}-C_{t\_v}^{np}\right)-(K_{up_{t}}\times C_{t\_v}^{np})-\left(K_{rel}\times C_{t\_v}^{np}\times V_{t\_v}\right)$$
    ○ Extravascular compartment
      (28) $$V_{t\_ev}\times \frac{dC_{t\_ev}^{np}}{dt}=(K_{up_{t}}\times C_{t\_ev}^{np})-\left(K_{rel}\times C_{t\_ev}^{np}\times V_{t\_ev}\right)$$
  ➢ Free stavudine
    ○ Vascular compartment
      (29) $$V_{t\_v}\times \frac{dC_{t\_v}}{dt}=Q_{t}\times \left(C_{p}-C_{t\_v}\right)+PS_{t}^{np}\times \left(\frac{C_{t\_ev}}{P_{t}^{np}}-C_{t\_v}\right)+\left(K_{rel}\times C_{t\_v}^{np}\times V_{t\_v}\right)$$
    ○ Extravascular compartment
      (30) $$V_{t\_ev}\times \frac{dC_{t\_ev}}{dt}=PS_{t}^{np}\times \left(C_{t\_v}-\frac{C_{t\_ev}}{P_{t}^{np}}\right)+(K_{rel}\times C_{t\_ev}^{np}\times V_{t\_ev})$$
      where subindices and superindices mean: *_b_*, brain; *_gt_*, general tissue; *_l_*, liver; *^np^*, gold nanoparticles based delivery system; *_p_*, plasma; *_s_*, spleen; *_t_*, thymus; and abbreviations mean: $C_{i\_v}^{np}\;\mathrm{and}\;C_{i\_ev}^{np}$ stavudine-AuNP vascular and extravascular concentration; *C_i_v_* and *C_i_ev_*, stavudine vascular and extravascular concentration; *Cl*, clearance; *K_rel_*, AuNP release rate constant; *K_up_*, AUNP uptake constant; $P_{i}^{np}$, partition coefficient when drug is administered with nanoparticles; $PS_{i}^{np}$ permeability-surface area coefficient when drug is administered with nanoparticles; *Q_i_*, blood flow; *V_i_*, compartment volume; *Vd*, distribution volume.

- Model Parameters

Model parameters have been obtained from literature, previous experiments, and computer optimisation.

Physiologic parameters of the PBPK model for rats (weight of 250 g), such as organ volumes, both total and vascular fraction, plasma flows and apparent permeability were the mean values found in the literature (Table 1) [22,23,24,25]. The *Cl* and *Vd* values used were obtained from a model-independent analysis of in vivo study results. The nanoparticle drug uptake rate constant (*K_up_*) was optimized by non-linear regression for each tissue (Table 1), using the value obtained in the in vitro uptake kinetic study in macrophages as the initial estimate. Similarly, the coefficient of permeability surface (PS) was also optimised for each tissue from the product of apparent permeability and organ flow.

The tissue/plasma partition coefficient (P) was calculated as the ratio of ${\mathrm{AUC}}_{\infty }^{0}$ estimated from the model-independent analysis of in vivo experiments in rats, except for general tissue, which was optimised. Finally, the release rate constant (*K_rel_*) value was taken from drug release study at physiological pH (pH 7.4), to be used both in vascular and extravascular compartments.

- Model validation

The predictive performance of the model was assessed by overlaying the simulated plasma and tissue concentration time profiles with the observed data. 

The overall predictability of the model was evaluated in terms of bias and precision from average-fold error (AFE) and absolute average-fold error (AAFE), respectively. Calculated AFE was considered acceptable if it was within a 2-fold error (0.5–2-fold) [26].

The equations used for the calculation of AFE and AAFE were the following:

(31) $$\mathrm{AFE}=10^{\frac{1}{n}\sum \log\left(\frac{PRED}{OBS}\right)}$$

(32) $$\mathrm{AAFE}=10^{\frac{1}{n}\sum |\log\left(\frac{PRED}{OBS}\right)|}$$

### 2.7. Softwares

The modelling and simulations of PBPK model, as well as non-compartmental analyses, were performed with Phoenix^®^ WinNonlin^®^ 64 (Version 7.0.0.2535, Certara, LP, Princeton, NJ, USA). Parameter estimation followed a naïve pooled strategy and a BFGS quasi-Newton algorithm. A statistical analysis was made with the SPSS v.20 software package and Graph Pad Prism 5.0 (Graph Pad software, San Diego, CA, USA).

## 3. Results

### 3.1. Development and Characterization of Stavudine Gold Nanoparticles

Drug loading efficacy was characterized for every batch of the stavudine nanocarriers, with a mean value of 67.2 ± 2.5%.

**Figure 4:**
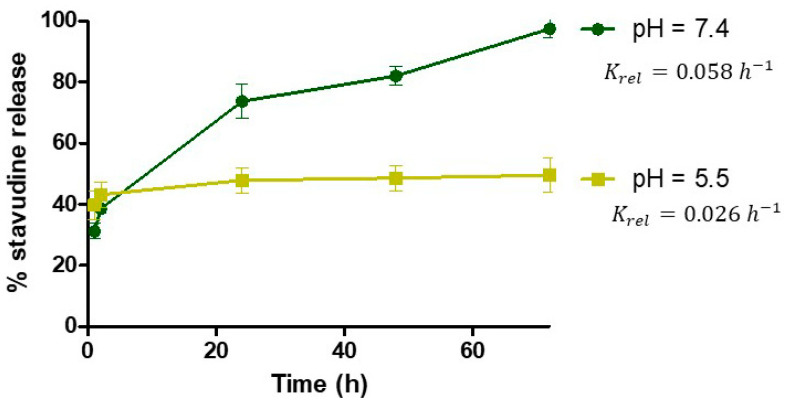
Release profile of stavudine from gold nanoparticles at pH 5.5 and 7.4 (in-house experiments). *K_rel_*: release rate constant for each pH.

The drug release profiles of stavudine nanocarriers, from in-house experiments, at pH 7.4 and 5.5 are shown in Figure 4. In both cases, there was a burst effect, followed by a sustained release of stavudine. However, under acidic conditions the release reached a plateau at 24 h, while under physiologic conditions, all the drug is released only after 72 h. 

According to the first order fitting, the estimated *K_rel_* is 0.058 h^−1^ at physiological pH (7.4) and 0.026 h^−1^ at acidic pH (5.5). 

### 3.2. Uptake Kinetics in Cells

**Figure 5:**
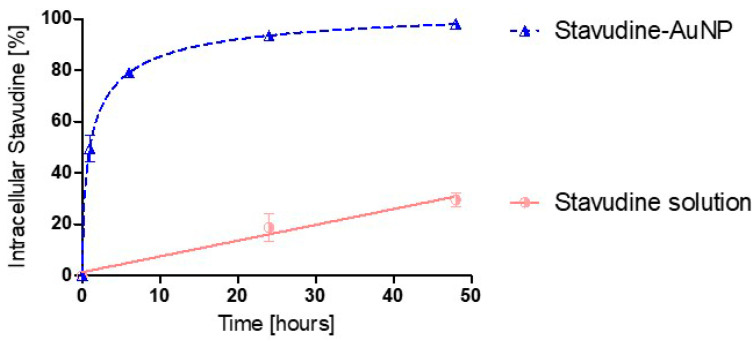
Uptake kinetic profile of stavudine percentage in cells (in-house experiments). *K_up_*: drug-nanocarrier uptake rate constant.

The in vitro results, from in-house experiments, showed that the percentage of intracellular stavudine concentration is much higher with gold nanoparticles as a delivery system than with the drug solution. In fact, stavudine-AuNP uptake follows a first order kinetics, with a *K_up_* of 0.564 h^−1^, while stavudine solution follows a zero-order kinetics, which is much slower (Figure 5).

### 3.3. In Vivo Pharmacokinetic Study 

**Figure 6:**
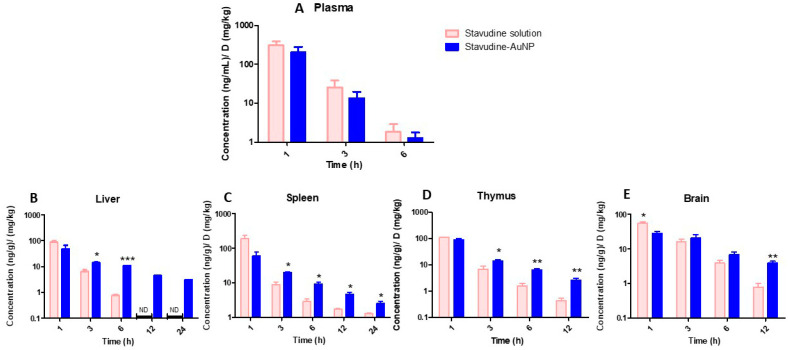
Normalised stavudine concentrations in each tissue (ng/g)/(mg/kg) at different times. (**A**), plasma; (**B**), liver; (**C**), spleen; (**D**), thymus; (E), brain. Significance levels: * *p* < 0.05; ** *p* < 0.005; *** *p* < 0.001.

Figure 6 shows in vivo stavudine concentrations standardised by dose in the studied tissues. Stavudine concentrations in plasma showed no statistically significant differences between the two groups (*p* > 0.05) (Figure 6). However, there were statistically significant differences (*p* < 0.05) in tissue stavudine concentrations in most of the analysed times (Figure 6).

### 3.4. Kinetic Analysis

#### 3.4.1. Model-Independent Analysis of In Vivo Study Results

The in vivo estimated model-independent parameters of ${\mathrm{AUC}}_{\infty }^{0}$, MRT and t_1/2_ are shown in Table 2. Moreover, *Cl* and *Vd* were 0.46 L/h and 1.84 L/kg for stavudine administered as a solution and 0.73 L/h and 2.92 L/kg for stavudine-AuNPs, respectively.

The sustained release of drug from nanoparticles leads, in all of studied tissues, to a higher MRT than with the stavudine solution. This increase is especially remarkable in the liver (Table 2). 

#### 3.4.2. PBPK Model

**Figure 7:**
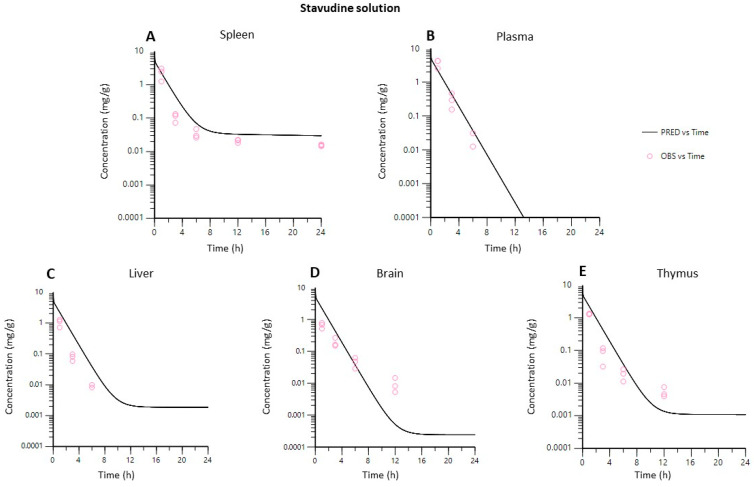
Observed vs. predicted concentrations in each tissue using the PBPK model for stavudine solution. (**A**), spleen; (**B**), plasma; (**C**), liver; (**D**), brain; (**E**), thymus.

**Figure 8:**
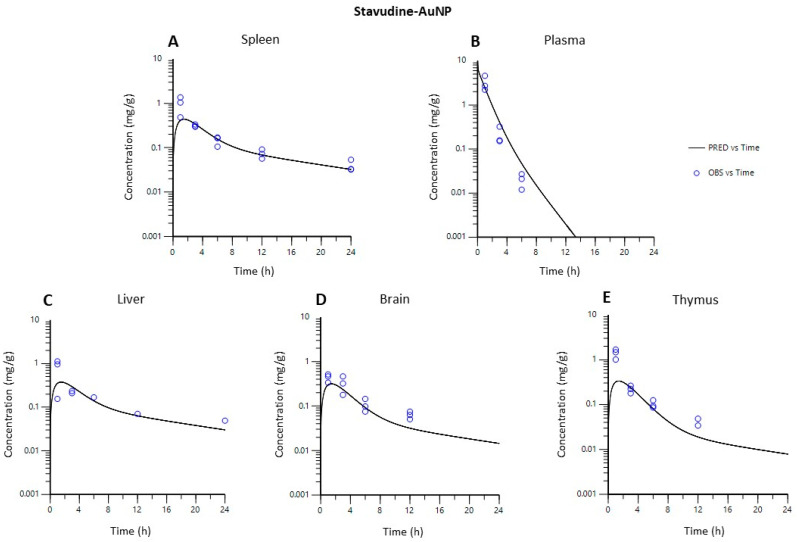
Observed vs. predicted concentrations in each tissue using the PBPK model for stavudine-AuNP. (**A**), spleen; (**B**), plasma; (**C**), liver; (**D**), brain; (**E**), thymus.

Figure 7 and Figure 8 show that both PBPK models developed described the in vivo data adequately in each of the tissues studied.

Bias and precision indices (AFE and AAFE) for the stavudine-AuNP predictions for each of the tissues were in all of the cases between 0.5 and 2.0 (Table 3).

**Figure 9:**
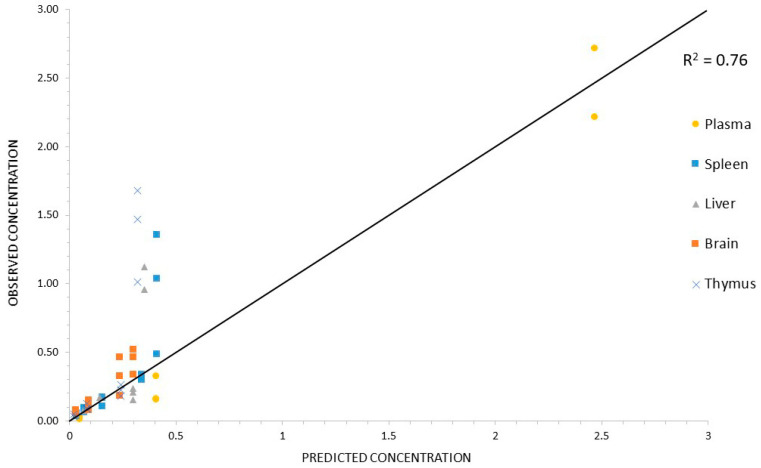
Correlation between observed vs. predicted concentrations for Stavudine-AuNP. Identity line (black line) was used as reference.

This good predictability can also be observed in Figure 9, which shows that the observed and predicted concentration values for stavudine-AuNP correlated, with a correlation coefficient of 0.76.

## 4. Discussion

Due to advances in computing capability and algorithms, regulatory agencies have considered in silico tools to promote drug research development and product design. Indeed, since the acceptance of PBPK simulation results by regulatory agencies began, there has been a meaningful increase in the number of publications, especially those related to the use of nanoparticles [6,27]. 

There are very few PBPK models in the literature about anti-HIV drugs or gold nanoparticles, and all of them are only for nanoparticle or drug solution administration [23,28]. However, this work is focused on the PBPK modelling of stavudine biodistribution after the administration of drug-gold nanoparticles.

Several in vitro and in vivo experiments were performed to address the lack of values in the literature, especially for parameters related to the gold nanoparticle delivery system.

Stavudine joins AuNPs with high efficacy. The stavudine molecule has amine groups which can bind to Au through weak covalent or electrostatic interactions [29]. In both cases, these weak bonds can easily be displaced by physiological ions. The presence of stavudine on the surface of AuNP was proven by the zeta potential change, in our previous study [16]. Although the exact binding mechanism is not determined in the current research, both types of bonds could be present, which would explain the initial burst effect and the sustained pH-dependent release (Figure 4). The *K_rel_* value at physiological pH has been selected for the PBPK model because the physiological environment is present in both the vascular and extravascular compartments. Only acidic pH is expected in later endosomes, but even if nanoparticles tend to accumulate in them, this cannot be modelled with the available data [30].

The in vitro studies have shown that the association with nanoparticles increases the stavudine uptake by macrophages (Figure 5). 

For in vivo experiments, the selection of the AuNP dose was based on the toxic concentrations studied by our group and others [16,31]. For the stavudine-AuNP dose calculation, the drug loading efficacy in the in vivo study was taken into account. The biodistribution results revealed that differences in plasma concentrations between the solution and nanoparticles were not statistically significant (*p* > 0.05) (Figure 6). However, stavudine tissue concentrations were higher after the administration of gold nanoparticles in most cases, especially at the last sampling hours, with statistically significant difference (Figure 6). 

These changes in biodistribution have a repercussion on the partition coefficient (P). The increase in P of stavudine, when gold nanoparticles are used, corresponds to the increase in the permeability, especially in the liver (Table 1). It is important to highlight that in all analysed tissues, the drug affinity towards tissues is higher with stavudine-AuNP (Table 1).

In addition to the targeting effect, the use of nanoparticles also leads to an increase in MRT and t_1/2_ of the drug in all of the tissues (Table 2). The increase in the MRT was especially relevant in important HIV reservoirs, such as the liver and spleen, but also in the thymus and brain. In fact, similar results have been shown with gold nanoparticles for other antiretroviral drugs [32].

In vivo model-independent pharmacokinetic parameters obtained are different from those published with other stavudine nanocarriers. The administration of lipid nanoparticles by i.v. route increased the ${\mathrm{AUC}}_{\infty }^{0}$, mainly in the blood and spleen, but drug concentrations in the brain and thymus were not detected [33]. On the other hand, other organic nanoparticles like polymeric ones need ligands like transferrin to increase drug concentrations in the brain in comparison with gold nanoparticles [34].

The parameters used for PBPK model building were obtained from the literature, in vitro and in vivo studies, and computer optimization. Tissues were modelled as permeability rate-limited, based on the fact that these kinetics occur with hydrophilic and larger molecules, like stavudine, and that in vitro study results revealed a very low cell drug uptake from the stavudine solution (Figure 5). Therefore, it was assumed that the non-facilitated diffusion through the lipidic vascular membrane would be hindered, and permeability across the vascular membrane becomes the limiting process. Nevertheless, the use of gold nanoparticles leads to an improvement in the stavudine permeability through the cellular membranes, as is shown by the PS estimated values (Table 1). 

The PBPK model gave a useful general picture of stavudine disposition in vivo with formulations, solutions, and gold-nanoparticles (Figure 7 and Figure 8).

Predicted drug tissue concentrations for stavudine AuNP show an appropriate correlation with observed ones, with the exception of some data at the initial times of the curve for the spleen, liver and thymus (Figure 9). The less acute fitting during the first hours after the administration has also been observed in other PBPK models with liposomes [35]. Moreover, a high variability in the data at this time has also been observed, especially in the liver and spleen. Thus, the lower quality of the data could limit the predicted capacity of the PBPK model for these data.

For the validation of concentration time measurements, summative metrics of bias and precision (AFE and AAFE) have been used as evaluations [36]. The AFE of the predicted concentrations was between 0.5 and 2 in all cases. For the stavudine-AuNP PBPK model, the AFE values of nearly 1 indicate a lack of bias associated with model predictions (Table 3).

Nevertheless, there were notable uncertainties in the modelling, and some limitations must be acknowledged. The heterogeneity in experimental design, small sample sizes and large variability in the pharmacokinetic results limit precise assessments of model accuracy in silico. It was noteworthy that the administration route modelled is not exactly the same as in the in vivo study, as has been previously explained [19], and that the exocytosis process of nanoparticles has not been considered in the model, due to the low exocytosis rate in the macrophages of anionic 40 nm gold nanoparticles [37]. Furthermore, more in vivo standardized biodistribution information, especially during the first hours, could help to improve the model’s predictions. However, despite these limitations, the developed model adequately described plasma and tissue concentrations after both stavudine solution and stavudine-AuNP administration.

Regardless of its limitations, the developed PBPK model allows a good prediction of tissue stavudine distribution when administered as gold-nanoparticles. According to all these results, in addition to these and previous studies carried out by our group [16], the gold nanoparticle-based stavudine delivery system developed has desirable characteristics of an HIV drug delivery system, such as: high loading efficacy, controlled release, and the capability to reach HIV target cells [8]. 

## 5. Conclusions

The PBPK model developed for the stavudine gold nanoparticle-based drug delivery system adequately described plasma and tissue stavudine concentrations in rats, with an average-fold error in all of the tissues between 0.5 and 2.0. The higher biodistribution of stavudine into HIV reservoirs and the modification of pharmacokinetic parameters such as the mean residence time (MRT) or half-life have also been properly described by this model. These changes are especially remarkable in the liver, which presents a higher partition coefficient (from 0.27 to 0.55) and higher MRT (from 1.28 to 5.67). Thus, stavudine-AuNP, which meets important characteristics for an HIV drug delivery system, such as drug payload, sustained release, and increased in vitro and in vivo drug concentrations into the cells and tissues, represents a novel and promising nanotechnological strategy for anti-HIV drugs, and the PBPK model approach may help with their pre-clinical extrapolation to other administration routes or species.

## Figures and Tables

**Table 1:** Physiologic and estimated pharmacokinetic parameters used in the PBPK model.

Tissue	Group	Flow (L/h)	V (L)	PS (L/h)	*K_up_* (h^−1^)	P
Plasma	Stavudine solution	3.09	-	-	-	-
	Stavudine-AuNP		-	-	0.56	-
Brain	Stavudine solution	0.08	1.5 × 10^−3^	3.75 × 10^−7^	-	0.36
	Stavudine-AuNP			0.17	7.9 × 10^−3^	0.46
Liver	Stavudine solution	0.60	1.2 × 10^−2^	2.65 × 10^−6^	-	0.27
	Stavudine-AuNP			14.4	0.15	0.55
Spleen	Stavudine solution	0.18	7.0 × 10^−4^	7.50 × 10^−6^	-	0.70
	Stavudine-AuNP			0.49	0.03	0.77
Thymus	Stavudine solution	0.02	5.0 × 10^−4^	9.62 × 10^−8^	-	0.28
	Stavudine-AuNP			0.10	1.00 × 10^−3^	0.61

**Table 2:** Model-independent pharmacokinetic parameters of stavudine solution and stavudine-AuNP groups estimated from in vivo study.

Tissue	Group	^1^ ${\mathrm{AUC}}_{\infty }^{0}$	MRT (h)	t_1/2_ (h)
Plasma	Stavudine solution	535	1.28	0.68
	Stavudine-AuNP	342	1.25	0.70
Thymus	Stavudine solution	193	1.51	1.54
	Stavudine-AuNP	209	2.69	2.15
Brain	Stavudine solution	146	2.54	1.84
	Stavudine-AuNP	159	5.77	3.81
Spleen	Stavudine solution	352	2.98	4.34
	Stavudine-AuNP	263	7.80	5.75
Liver	Stavudine solution	151	1.28	0.74
	Stavudine-AuNP	189	5.67	4.50

^1^ ${\mathrm{AUC}}_{\infty }^{0}$ units: Plasma: (ng*h/mL)/(mg/kg); tissues: ((ng*h/g)/(mg/kg).

**Table 3:** Prediction errors for stavudine-AuNP PBPK model in plasma and tissues.

Indices	Plasma	Brain	Liver	Spleen	Thymus
AFE	1.61	0.71	0.81	0.85	0.57
AAFE	1.89	1.53	1.68	1.32	1.88

AFE, average-fold error; AAFE, absolute average-fold error.

## Data Availability

The data presented in this study are available on request from the corresponding author.
